# Beyond metrics: relational dynamics and impacts of TB community funding

**DOI:** 10.3389/fpubh.2026.1851740

**Published:** 2026-06-16

**Authors:** Anna Versfeld, Caoimhe Smyth, Viorel Soltan, James Malar, Augusta Baiden, Liliana Caraulan

**Affiliations:** 1 Department of Sociology and Anthropology, Nelson Mandela University, Gqeberha, South Africa; 2 Stop TB Partnership, Geneva, Switzerland

**Keywords:** community engagement, funding, stigma, TB community, tuberculosis

## Abstract

Global funding for tuberculosis (TB) falls far short of estimated needs, often prioritizing commodities and service delivery. TB communities, including people affected by TB, remain underfunded and underrepresented in programme planning. Despite this TB-affected communities have, over the past decade, driven significant progress in becoming recognized actors in national and global TB responses. These gains have been largely supported by the Stop TB Partnership Challenge Facility for Civil Society (CFCS), a funding mechanism which supports people affected by TB and community organizations and their partnerships with National TB Programmes. This model now faces a critical funding shortage. This analysis draws on final reports from 99 country-specific projects funded under CFCS 12 (2023–2025). In final reporting partners responded to three open-ended reflection questions on unexpected impacts, challenges, and lessons learned in. Responses were analyzed inductively using NVivo 12, with themes iteratively developed. Three key relational themes emerged. First, TB stigma was reported not only as a barrier to care but also as an operational barrier to programme delivery. Second, the value of meaningful community engagement and collaboration were identified as the most important lesson from programme implementation. Third, expanded recognition and influence were the most important unexpected outcome of programme delivery. These reflections highlight the relational challenges and value of TB community funding, directing us to consider what is included in funding considerations and project planning, while also pointing toward how much—beyond programmatic outcomes—will be lost if funding is reduced further.

## Introduction

Before the funding shocks of 2025, annual global financing for the tuberculosis (TB) response was approximately $5.7 billion—far below the estimated $22 billion required to end the epidemic (1) with donor-dependent activities such as community outreach and engagement particularly vulnerable to cuts (2).

The dramatic changes to the global health funding landscape in 2025 have further deepened this already severe shortfall (2). In this context, available resources are often directed toward essential commodities, such as diagnostics and medication, and core needs such as service delivery staff and infrastructure. TB communities are—in contrast to their HIV counterparts—underfunded and less established, and lived experience remains at the margins of TB programming and planning.

The Stop TB Partnership Challenge Facility for Civil Society (CFCS) grants mechanism is the only targeted mechanism funding affected community partners to implement TB projects. The CFCS model is based on the recognition that community actors, including affected TB networks, hold critical expertise derived from lived experience. It therefore prioritizes community leadership, capacity strengthening, and the expansion of community influence across the TB response. Programmatic focus areas cover expanding reach to underserved populations; increasing awareness and uptake of TB innovations; strengthening accountability and quality of care; and supporting the recognition, institutionalization, and sustainability of community systems.

As the global TB funding crisis takes hold, CFCS is at risk. TBpeople—a global network of affected people with 30 country chapters— raised the alarm about the potential consequences through a petition signed by over 11,000 people and a joint letter to the Global Fund from more than 70 CFCS partners. Dramatic reductions in TB community funding mechanisms raise an important question: What does CFCS deliver beyond funding, especially in transforming the role of communities within the TB response, and what will be lost if it is allowed to disappear?

CFCS partners are required to develop and report on coherent project plans with measurable indicators to assess the extent to which objectives are met. As a grants management team working across multiple grant cycles, our engagement with implementing partners revealed insights that extended beyond formal reporting, surfacing critical challenges as well as valued outcomes not adequately captured within standard metrics and frameworks. In the CFCS Round 12 (2023–2025) final grant reports, we therefore introduced additional sections to capture open-ended reflections. Partners were invited to reflect on challenges encountered during implementation, unanticipated positive outcomes, and lessons to inform future work. This piece goes some way to answering that question by drawing on a thematic analysis of those qualitative reflections from CFCS 12 country-level project reports.

The CFCS 12 projects were all half way through implementation when shifts in donor priorities altered the funding context in which they were being delivered, over and above discussions about the impact of these changes on work, three key themes emerged as primary concerns the analysis and hold broader relevance for ongoing funding and programmatic work. Firstly, stigma emerges not only as a barrier to care, but also a systemic constraint affecting programme delivery. Secondly, community engagement and collaboration emerged as central to project success yet were frequently under-resourced. Thirdly project implementation led to increased influence of community actors within the TB response, representing a significant and highly valued by-product that extended beyond project objectives and sustained their capacity to influence TB efforts beyond project implementation.

These reflections provide insight into dimensions of TB programme implementation that are rarely visible in conventional reporting metrics. While community funding mechanisms such as CFCS are often evaluated in terms of the interventions they deliver, the experiences of implementing organizations suggest that the impact extends further, shaping relationships, trust, and influence across health systems, national tuberculosis programmes and communities. Understanding these relational dynamics is particularly important at a time when global TB financing is shrinking and programmes that support community systems are increasingly experiencing funding cuts. The themes below therefore examine what these projects reveal about the relational work that grant mechanisms enable, and what will be lost if such funding declines.

## Methods

This analysis draws on final reports submitted by partners participating in Round 12 of the Stop TB Partnership's Challenge Facility for Civil Society (CFCS), implemented between late 2023 and mid-2025. CFCS 12 provided grants to 111 organizations. This included grants 100 country-specific projects implemented across 38 countries, nine grants for regional projects, and two grants for global work.

Each project partner was required to submit a final report on project completion as part of routine programme processes. After reporting against predefined objectives and indicators, partners were asked to reflect on their work through responding to three open-ended questions:

- Please tell us about any unexpected challenges and how you responded to these.
- Please describe any additional lessons learned through the project and why these are important.
- Please tell us about any other ways you have noticed your project having a positive impact, even if these are not directly related to your planned activities or expected outcomes.

This paper draws on these reflections from the 99/100 country-specific project reports that were available in November 2025, but excludes the regional and country grant reports, which were aligned with a different set of programmatic objectives. Answers were analyzed inductively using NVivo12 [Lumivero (Formerly QSR International), Denver, Colodaro, USA], with codes iteratively developed and organized into an analytic framework as themes emerged. Coding was conducted by a single analyst, with emerging themes reviewed, sense-checked between the writing team, and findings checked against an exit questionnaire filled by 71 country-based partners in July 2025. A descriptive count of reports referencing each theme was also conducted to map distribution across organizations. A theme was counted where it was mentioned at least once in a report.

The writing team consist of the CFCS grants management team, and programme leadership. This sets up a particular relationship to the data. We managed our own potential biases by ensuring that every assertion was backed by unambiguous data from the reports, incorporating both positive and negative reflections, and using direct quotes to represent perspectives.

## Stigma as an operational barrier to programme delivery

TB stigma is one of the key barriers to care access and a significant personal challenge for people affected by TB. This is well-established in literature, including systematic reviews (3, 4) and reinforced by national stigma assessments (5) and OneImpact community led monitoring, supported by Stop TB Partnership. Reflections from CFCS partner reports highlight a further consideration: TB stigma is also a barrier to programme delivery. Despite the absence of explicit prompts, stigma emerged as an unanticipated constraint during project implementation over a quarter of partners (23/99). The only challenge reported more frequently was the change in donor priorities which impacted all CFCS projects.

TB stigma limited uptake of services such as community screening, contact tracing, and enrolment in social support programmes, especially in cases of drug-resistant TB. TB stigma, and fear of association with the disease, meant that community leaders were not always willing to acknowledge TB in their communities or publicly endorse projects. TB stigma made recruitment of management and implementation teams more difficult; and it undermined TB survivors' willingness to serve as peers and to have their stories featured in advocacy materials. These greater difficulties in engaging people to publicly be associated with the projects were reported even by partners experienced in the TB sector. In some cases, stigma-reduction interventions were initially blocked by healthcare providers concerned about exposing their own discriminatory practices. Projects also had to provide rapid support for people with TB experiencing discrimination. In each of these cases, organizations had to invest additional time and resources for planned interventions.

CFCS has played a crucial role in highlighting how TB stigma manifests across individuals, families, healthcare facilities, and workplaces through stigma assessments and community-led monitoring (5). This analysis extends that perspective by emphasizing stigma as an operational concern. Project proposals and designs need to account explicitly for the scale and forms of stigma in implementation contexts and anticipate its effects on staffing, safeguarding, and programme delivery. Addressing stigma is relevant to individual experience and human rights, but also as a matter of programme efficacy.

## The importance of working together: community engagement and collaboration

Almost half (44/99) of the partners reported that the value of community engagement was one of the most important lessons from their CFCS 12 work. This must be situated in a context of widespread experiences of exclusion. While community engagement is a basic pillar of effective TB implementation work (6), communities experiencing the highest TB burden are often systematically excluded from TB programme planning, implementation, evaluation, and research (7, 8).

Community engagement described by the partners extended to people affected by TB and DR-TB, religious and community leaders, families, health service providers, and other civil society organizations. It was valued for the local ownership it engendered. As one partner reflected, “When village leaders, health cadres, and local institutions are meaningfully involved from the start, through participatory planning, capacity-building, and shared decision-making, they become active champions of TB response efforts.”

Engagement and inclusion of people affected by TB and DR-TB was described as valuable for influencing decision-makers and peers, as lived experience brings authority and persuasive power to messaging. Engagement was also valued for the way it supported cultural sensitivity and adaptation of interventions to local context. This was reported to be particularly important in high-stigma settings where organizations had to establish their own legitimacy, and justify the work being done. These reflections highlight what becomes possible when meaningful engagement is prioritized—and implicitly, what may be lost when it is treated as optional.

Partners also pointed to conditions that made engagement effective: involvement from the outset (including in planning), sufficient time for trust-building, and sustained capacity strengthening and mentorship. In addition, Stop TB Partnership requires and supports the engagement of National TB Programmes as an integrated aspect of all interventions. This is reflected in collaborative approaches such as the joint rollout of stigma and key and vulnerable population assessments, and the implementation of OneImpact, where communities and National TB Programmes work together to identify missed populations, generate and interpret evidence, and support the integration of findings, recommendations, and costed actions into national strategic plans. This all suggests that engagement, like stigma mitigation, must be embedded in planning and budgeting processes rather than appended as an activity.

Closely related to community engagement, almost half (39/99) of the partners emphasized that the project work had highlighted the importance of collaboration—working together with other CFCS partners, civil society networks, health sector actors, government departments (including health, labor, and criminal justice), academia, and the private sector—for strengthening the TB response.

Collaboration between CFCS partners was valued because sharing skills, knowledge, and activities expanded the areas of work and strengthened impact. Partners described how this expanded the psychosocial services they were able to offer people affected by TB, which was especially important for people with DR-TB. Collaboration with organizations not traditionally engaged in TB—including those focused on HIV, disabilities, and supporting the health and wellbeing of women and girls—was valued for the way that it extended the reach of TB services to underserved populations. Partners also reflected that working with organizations not funded by CFCS meant that interventions were less tied to the project period, as the work initiated could be continued by other organizations after the grant was closed.

Established relationships with other civil society organizations also served to foster resilience. As funding priorities shifted, organizations were able to pool resources, allowing them to continue crucial TB services and survive the sudden block on funding. At the same time, some partners noted that funding scarcity fostered competition, undermining collective efforts. With shrinking global health funding, collaboration becomes both increasingly vital and increasingly fragile.

These reflections demonstrate the importance of deliberately investing in community engagement and cross-sector collaboration. People affected by TB should participate in matters that affect them (9). Furthermore, excluding high-burden communities from planning and implementation risks weak networks, lower trust, and reduced legitimacy, which in turn limits programme reach and uptake.

Engagement and collaboration require time, resources, and deliberate planning; when these are insufficient, opportunities to strengthen local ownership, build advocacy capacity, and foster resilient partnerships are lost. Conversely, when prioritized, these relational investments create durable social capital, expand influence for people affected by TB, and increase organizational and programme resilience in the face of funding shocks. Funding models and programme design must therefore recognize and support relational work as a core component of TB responses, rather than treating it as optional or ancillary, to ensure both immediate effectiveness and long-term sustainability.

## Unexpected impact: expanded recognition and influence

While the effects of TB stigma on programme implementation were the most commonly described unexpected barrier to project implementation, and the importance of community engagement and collaboration was the most important lesson, the resulting expanded recognition and influence were described as the most important unexpected positive outcomes of project implementation, noted by 55/99 organizations. This was especially widely reported in the organizations implementing OneImpact Community-led Monitoring.

With the government sector, recognition and influence manifested as participation in technical government meetings, inclusion in accountability and oversight bodies such as Global Fund Country Coordinating Mechanisms, and inclusion in United Nations High Level Meeting discussions and delegations, and involvement in broader public health initiatives, such as immunization and nutrition campaigns. Official recognition as a Global Fund sub-subrecipient was also noted, for example by an organization in Indonesia.

This inclusion had a double benefit: partners started to influence decision-making through contributing their own expertise, while also contributing skills and social capital to government-led activities.

Within organizations, partners described the increased recognition and respect for TB survivors as peers, organizers and advocates. As a partner from Nepal reflected, “Survivors, once reluctant to speak publicly, have now become vocal advocates in their communities, addressing broader social issues such as mental health, gender-based discrimination, the need for social protection and access to education.” In several projects, survivors started to be included in broader health and community committees, extending their influence beyond TB, for example in TB/HIV integrated community-led monitoring initiatives.

Eight partners also described how the project enhanced the self-confidence, skills, and leadership of women community health workers. A partner from Tanzania reported, “The 40 empowered women community health workers have become respected advocates and leaders in their localities, and are now often consulted on TB-related and gender-sensitive health matters.” TB project implementation can therefore have a broader impact by addressing gender inequalities and empowering women as leaders in health and community settings.

These project reflections point toward durable shifts in influence and leadership, both within communities and in broader health systems, that can result from investing in community-led TB projects. Funders and programme designers should value and support relational and empowerment outcomes as core components of TB responses. They enhance the long-term resilience, reach, and sustainability of interventions (Table 1).

**Table 1:** Thematic quotes.

Thematic frequencies and demonstrative quotes
Stigma as an operational barrier to programme delivery
**Report count:** 23/99 (23%) organizations
“Another challenge is the stigma surrounding DR-TB, which can discourage patients from seeking care or participating in support programs.” “Despite awareness efforts, many [healthcare workers] were reluctant to participate in screenings due to the stigma associated with TB diagnosis.” “One unexpected challenge was the initial hesitation of traditional leaders and community members to engage in discussions about TB, largely due to deeply ingrained stigma and fear of social exclusion.” “We also faced social challenges, especially when looking for TB survivors who were willing to share their stories in a public podcast. The fear of stigma was a big obstacle.”
The value of community engagement as a key implementation lesson
**Report count:** 44/99 (44%) organizations
“A key lesson learned is the importance of building trust over time within marginalized communities. Effective TB response requires more than information—it needs local champions, culturally sensitive communication, and consistent engagement.” “Building trust and involving the community early and consistently in project planning and implementation fosters ownership and long-term commitment to TB care.” “One of the most important lessons learned was that when TB-affected communities are meaningfully involved in designing and implementing interventions, they take ownership of both the process and the outcomes.” “An important lesson is the value of early and sustained engagement with traditional and religious leaders, especially in settings where they hold significant social influence. Their support proved essential in legitimizing the [CLM] platform and encouraging openness to sensitive topics like stigma, discrimination, and accountability.”
The importance of collaboration as a key implementation lesson
**Report count:** 39% (39/99) organizations
“The project has highlighted the importance of strengthened collaboration between community players, health structures and local authorities to guarantee sustainable access to care, particularly in hard-to-reach areas.” “Another important lesson was the value of engaging [civil society organizations] from non-health sectors. Their involvement significantly expanded outreach to key and vulnerable populations who are often underserved by the traditional health system.” “By working with social services, village development agencies, religious leaders, academia, and philanthropic groups, we were able to provide more comprehensive and dignified support to TB-affected households.” “Finally, collaboration among CFCS grantees enriched technical approaches and created stronger national coherence around community-led TB accountability.” “Strategic collaboration with the National TB Programme, legal support groups, and media organizations helped transform individual cases into system-wide action.”
Expanded recognition and influence as a key unexpected positive outcomes of project implementation
**Report count:** 55/99 (56%) organizations
“One of the most significant and encouraging unintended outcomes of the project has been the increased recognition of the emerging TB community as a legitimate stakeholder in national policy dialogue.” “…we observed a noticeable shift in attitudes: government representatives began proactively inviting CSOs to participate in technical meetings and consultations and service improvements.” “The project also enhanced trust between the community and the formal health system… This increased trust is already facilitating better participation in other government health initiatives, such as immunization campaigns and nutrition programs” “One key outcome was the emergence of new community leaders, particularly among DR-TB survivors and youth who began taking initiative in mobilizing others, facilitating dialogue sessions, and advocating for improved TB services at local levels.” “Several TB Champions gained recognition beyond TB related work and were invited to participate in local health and social welfare committees. This has positioned them as key voices for marginalized communities.” “Women who had previously been hesitant to speak out began taking leadership roles in their communities, advocating for improved services and supporting peers facing health or social challenges.”

## Discussion

Public health funding is typically structured around defined plans with measurable outputs, with TB grants often prioritizing diagnosis and treatment targets. In contrast, CFCS focuses on strengthening TB community actors as a foundation for improved health systems and TB programming. As a grants support team, we have observed the evolution of CFCS-supported organizations over successive funding cycles, noting shifts in the positioning, capacity and influence of TB community actors. These shifts are most evident where financial support is coupled with institutional strengthening and enhanced convening power. They have not been systematically measured, nor has their contribution to broader systems change been fully captured.

Reflections from one round of CFCS partners demonstrate the processes through which these changes occur: major barriers such as stigma come into focus, collaboration and engagement are strengthened, and community influence expands. The value of these grants therefore extends beyond quantifiable outputs, accruing over time through sustained relational change and continuing beyond the lifespan of individual projects. This is particularly critical in a context of declining TB funding, which places increasing demands on community partners while simultaneously constraining the resources needed to meet them. Relational work takes time and resources, and is critical for strengthening project delivery, building organizational resilience and expanding impact. It generates social capital, broadens organizational focus, and increases influence, often persisting beyond the lifespan of specific interventions. This challenges conventional ideas of sustainability: even when programme activities cease, their effects may endure through sustained engagement and influence. At this time, when we are faced with shifting health infrastructures, the challenges of drug-resistant TB, the health security challenges of antimicrobial resistance, and the need for widespread vaccine sensitization and demand creation, these gains are critical.

In making these assertions, we note a few limitations. Firstly, the findings are based on self-reported project reflections provided by partners through a grant management system, which may have encouraged over-emphasis on positive outcomes, especially in a resource-constrained environment. Secondly, while the findings suggest that CFCS funding contributed to outcomes such as increased influence of TB-affected communities, the qualitative and retrospective nature of the data does not allow causal attribution, and there may have been other influences to the reported changes, which were also not externally verified. Finally, CFCS is a unique funding mechanism, and the findings presented here may not be transferable to other contexts and funding structures. The flip side of this is that the nature of the CFCS grants mechanism may provide insight into how best to foster relational growth through grant provision.

## Conclusion

Our findings of important relational changes resulting from project support—even through one grant cycle—are not intended to undermine the value of sustained, long-term funding. Indeed, CFCS experience demonstrates that sustained investment is associated with stronger community voice and influence at higher levels of decision-making. Rather, these reflections highlight the less acknowledged impact of TB community funding and support and suggest that as budgets shrink and CFCS and similar mechanisms come under pressure, we stand to lose more than conventional metrics routinely reveal.

## Data Availability

The deidentified data supporting the conclusions of this article will be made available by the authors, without undue reservation.
